# (Bio)Electroanalysis of Tetracyclines: Recent Developments

**DOI:** 10.3390/bios15020101

**Published:** 2025-02-11

**Authors:** Maria Madej, Paweł Knihnicki, Radosław Porada, Jolanta Kochana

**Affiliations:** Department of Analytical Chemistry, Faculty of Chemistry, Jagiellonian University, Gronostajowa 2, 30-387 Kraków, Poland; marysia.madej@uj.edu.pl (M.M.); pawel.knihnicki@uj.edu.pl (P.K.); radoslaw.porada@uj.edu.pl (R.P.)

**Keywords:** antibiotic resistance (ABR), tetracycline, biosensor, electroanalysis, photoelectrochemical, aptamer

## Abstract

Tetracyclines (TCs) are antibiotics used extensively in medicine, veterinary science, and animal husbandry. Their overuse and the widespread presence of their residues in the environment contribute to intensifying the phenomenon of antibiotic resistance (ABR). The efforts are being made to reduce the spread of antibiotics and control the phenomenon of ABR, and one of the key methods is monitoring the presence of antibiotic residues in the environment and food of animal origin. Herein, we provide the overview of the recent developments in electrochemical (bio)sensing of tetracyclines in different types of samples. The review presents a comprehensive view of such aspects of the practical (bio)sensor application as sample preparation, the reusability of (bio)sensors, and the possibility of determining antibiotics at levels required by regulations. Advances, existing challenges, and future trends in the development of novel (bio)electrochemical methods of tetracycline quantification were discussed.

## 1. Introduction

Starting with the discovery of penicillin in 1929, antibiotics have emerged as a powerful tool against bacterial diseases. Therapy involving antimicrobial drugs was one of the most significant medical breakthroughs of the 20th century [1]. They are widely utilized in human and veterinary medicine, improving the health of both humans and animals. However, misuses and overuses of antibiotics over the last decades have led not only to environmental pollution, but also to allergies, antibiotic resistance, and superinfections in humans [2]. The problem of bacterial resistance against antibiotics (ABR), being a result of excessive use and exposure to antibiotics, is presently a global issue that needs decisive, consistent actions. Tetracyclines (TCs) are one group of antibiotics that contribute significantly to this problem.

**Figure 1:**
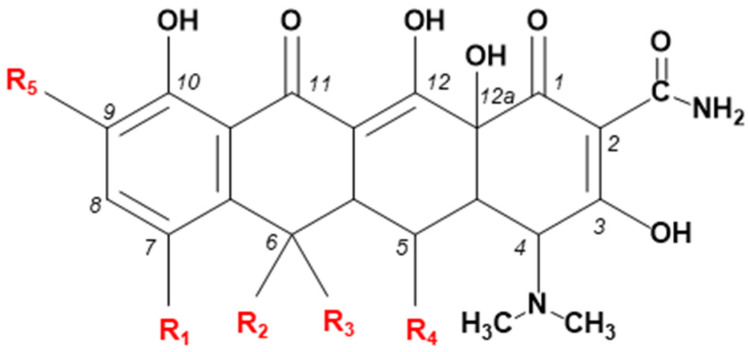
Schematic structure of antibiotics of the tetracycline group. Reproduced under the terms of the CC-BY license from Ref. [4] [*Pharmaceutics*].

Tetracyclines are a group of antibiotics that exhibit broad-spectrum activity against Gram-positive and Gram-negative bacterial pathogens. They are also employed to fight infections arising from a protozoan parasite and intracellular organisms [3]. Their high activity against Gram-positive and Gram-negative bacteria is based on the inhibition of protein synthesis by binding to a 30S ribosomal bacteria subunit [1]. TCs exhibit both bacteriostatic and bactericidal activity and are lethal to bacteria only in high concentrations. Their structure is characterized by the common backbone, a naphthacene carboxamide ring system, and their analogues differ in the substituents at positions C5, C6, C7, and C9 [4,5], as shown in Figure 1.

The discovery and development of tetracycline antibiotics can be considered in three stages. The first tetracycline was discovered in the late 1940s as a fermentation product of the golden-colored soil bacterium *Streptomyces aureofaciens* and was named aureomycin (AUR; chlortetracycline) [4].

Other representatives of the first generation of TCs, including oxytetracycline (OXY) and tetracycline (TET), were also natural substances [3]. The semisynthetic tetracyclines of the second generation, represented by doxycycline (DOX), lymecycline, meclocycline, methacycline, minocycline, and rolitetracycline, were obtained by modifying the side chains of first-generation antibiotics. They showed less toxicity and broader antimicrobial activity. The third generation of TCs includes semisynthetic and synthetic compounds. They demonstrated a considerable enhancement in antibiotic activity and pharmacological properties. Their antibacterial spectrum covers, among others, bacterial strains resistant to older generations of tetracyclines. Nowadays, they are used as first-line medicines for infectious diseases, such as mycoplasma pneumonia and brucellosis [3]. Representatives of this group are tigecycline, omadacycline, eravacycline, and sarecycline [4].

Tetracyclines are extensively used to treat humans and animals. Tetracycline is known to be the second most used antibiotic worldwide to treat bacterial infections [6]. Due to the low cost of production, wide availability, and broad spectrum of activity, TET is the most frequently utilized antibiotic in the animal breeding industry for protective and curative reasons. It is also often used in animal feed to enhance growth and feeding efficiency [7]. In 2019, for example, tetracyclines accounted for as much as 32% of total antibiotic sales [8]. However, tetracycline is metabolized in humans and animals only to a minor extent: in humans, about 60% of the dose taken, and in animals 17–80% of the drug, remains unmetabolized and is excreted in the urine and feces [6,8,9]. This results in the ubiquitous presence of tetracycline in the environment in soil, surface, ground water, and, due to insufficiently efficient water treatment procedures, even in drinking water. Taking into account the strong sorption capacity of soil, sediments, sludge, and manure towards TCs, their good water solubility, and environmental persistence, TCs are considered to be hazardous pollutants [6,10]. They contribute to the huge global problem of the persistence and biological activity of antibiotics that enter the environment [9]. It has been proven that the pervasive presence of antibiotics in the environment causes the development of antibiotic-resistant bacterial strains. The development of TCs-resistant microorganisms may occur in several ways [11]. Bacteria can synthesize enzymes that modify or degrade antimicrobial agents, leading to a reduction in their activity or even complete inactivity. In addition, bacterial proteins that are targets of the antibacterial action may change, blocking the effect of the antibiotic. A reduction in the permeability of the membrane to antibiotics may hinder the transport of the drug inside the bacterial cell. In turn, the efflux pump phenomenon may result in the effective elimination of the antibiotic out of the bacterial cell. Since resistant microorganisms can be transmitted between individuals through direct contact or indirectly through the exchange of resistant genes in the environment [9], this may lead to tetracyclines being ineffective in the treatment of certain bacterial infections. Long-term exposure of living organisms to TCs endangers their health through possible toxicity. Harmful effects of TCs may manifest as allergic reactions, renal dysfunctions, disturbance of natural intestinal microflora, intracranial hypertension, and skin infections. This may also be attributed to the consumption of animal-derived foods, since the result of the use of tetracyclines in animal husbandry is the presence of antibiotic residues in food products. To protect human health, the Food and Agriculture Organization, World Health Organization, and the European Union have issued regulations on the maximum TCs content in certain food products; for example, the maximum residue limit (MRL) is 100 μg/kg (0.1 ng/μL) in milk and eggs and 10 μg/kg (0.01 ng/μL) in honey [6,12]. The Joint Expert Committee on Food Additives suggests a maximum daily intake of tetracyclines of 30 μg/(kgbw·day) [6].

**Figure 2:**
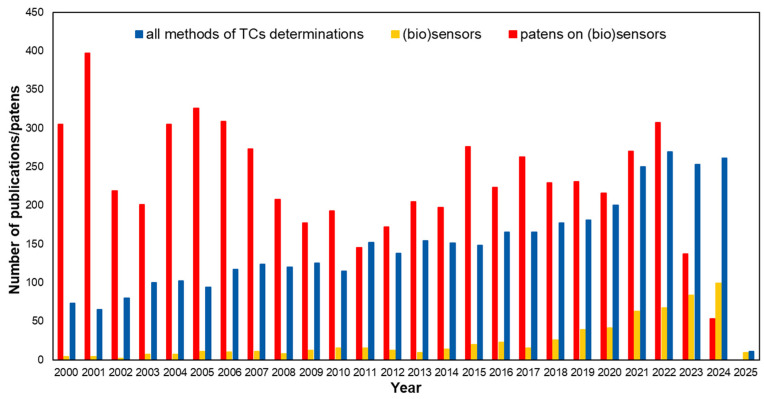
Chronological development of methods and patens for TCs assay between 2000 and 2025 (based on Scopus and Google Patents, 15 January 2025).

The issue of the ubiquity of antibiotics in the environment and animal-derived foods, disturbing reports of escalating antibiotic resistance phenomenon, as well as the discovery of new strains of bacteria resistant to all known antibiotics, are being undertaken by many researchers [13,14]. The efforts are being made to reduce the spread of antibiotics and control the phenomenon of ABR, and one of the key measures is monitoring the presence of antibiotic residues in the environment and foods of animal origin. Many analytical strategies have been proposed to achieve this goal. Among the sophisticated instrumental techniques, chromatographic ones coupled to ultra-sensitive detectors, such as liquid chromatography—high resolution mass spectrometry (LC/HRMS) or high-performance liquid chromatography—tandem mass spectrometry (HPLC/MS-MS), are the most frequently employed [15,16]. They provide the potential for sensitive and selective determination at low analyte concentrations; however, they require sophisticated equipment, skilled personnel, and a time-consuming step of preparing samples for analysis. The need for precise and accurate quantification and monitoring of TCs with simple and rapid procedures has resulted in the intensive development of new approaches based on the use of sensors and biosensors. Figure 2 shows the dynamics of research regarding methods for the determination of tetracyclines over the last 25 years. The graph is based on the number of publications relating to all methods of TCs assay and publications on the sensors developed, as well as the number of patents on (bio)sensors for TCs determination. It is clear that research into the development of sensors for TCs determination accounts for an increasing proportion of all reported methods for its determination. (Bio)sensors with optical and electrochemical transducers have been developed more often.

The objective of this manuscript is to review, evaluate, and summarize the progress of recent years in the development of (bio)electroanalytical procedures for the determination of antibiotics of the tetracycline group. Several review articles have recently been published, in part related to the (bio)electrochemical determination of tetracyclines. Zheng et al. presented ultrasensitive sensors and biosensors constructed for the determination of antibiotics in environmental samples [17]. The application of biosensors for food safety verification, taking into account the presence of, among others, antibiotic residues, was reported by Meliana et al. [18]. Singh et al. discussed the electrochemical biosensors for the quantification of antibiotics in milk [19], while Raykova et al. described the real-time analyses of milk for the determination of TCs using electrochemical methodology [8]. Sensors and biosensors with electrochemical transducers based on carbon electrode materials designated for the determination of tetracyclines were presented by Hermouche et al. [20], and Aihaiti et al. summarized electrochemical quantifications of antibiotics based on carbon nanocomposites [2].

To the best of our knowledge, there are no papers focused on electrochemical sensors and biosensors used in the analysis of tetracyclines in various types of samples.

In our review, the manuscripts were classified by the type of sample analyzed. The highest number of reported (bio)sensors concerned the determination of TCs in animal-derived food products, particularly in milk. A smaller group includes publications dealing with analysis of environmental and pharmaceutical samples. Taking into account the practical aspects, special attention was paid to the sample preparation and ability to regenerate the (bio)sensor and reuse it. The possibility of carrying out analyses at concentration levels indicated by regulations as permissible limits of antibiotic concentrations (content) in a given type of sample was also verified. The electroanalytical parameters of developed approaches are summarized in Table 1, Table 2 and Table 3, whereas the text focuses on presenting the most interesting, in the authors’ opinions, reports. In conclusion, achievements, strengths and limitations, today’s challenges, and future trends in the development of (bio)electrochemical approaches for TCs determination have also been discussed. This review covers articles published since 2022.

## 2. Reported Mechanisms of (Bio)Electrochemical Sensing of TCs

Among the reports relating to TCs determination, those referring to aptasensors dominate. Aptasensors constitute a broad group of biosensors enabling the determination of analytes in a sensitive and specific manner, even in samples with very complex matrices. Aptamers (Apt) are artificial DNA or RNA oligonucleotides that have a strong affinity for low-molecular-weight compounds and proteins. They are obtained using the SELEX (systematic evolution of ligands by exponential enrichment) method. In contrast to antibodies, aptamers are chemically synthesized, which makes them more stable, easier to modify, and simpler to immobilize on solid supports. Moreover, after use for biological recognition, aptamers can be regenerated without losing their selectivity [21]. The developed aptasensors mainly used electrochemical detection, and there were also examples of employing photoelectrochemical (PEC) and electrochemiluminescence (ECL) detection. Two aptasensors using dual electrochemical/fluorescence dual-mode detection were also developed.

Electrochemiluminescence involves the emission of light triggered by electrochemical reactions. This phenomenon can be used as a sensing strategy in (bio)sensors if stable and efficient materials with luminescent properties are used for its construction. Electrochemically luminescent materials undergo redox reactions, resulting in electron transfer and the formation of the excited state. When returning to the ground state, they emit a light signal. In order to induce luminescence of the electrode, composite potassium persulfate is commonly used. As a result of potential sweep, the S_2_O_8_^2−^ ions undergo reduction, accompanied by the formation of the sulfate radical anion (SO_4_^•−^), which introduces electrons to the highest occupied molecular orbital of luminescent material. If a biomolecule such as DNA or an aptamer is immobilized on the surface of such a material, its ECL intensity declines proportionally to the concentration of the analyte that interacts with the biological element [22].

**Figure 3:**
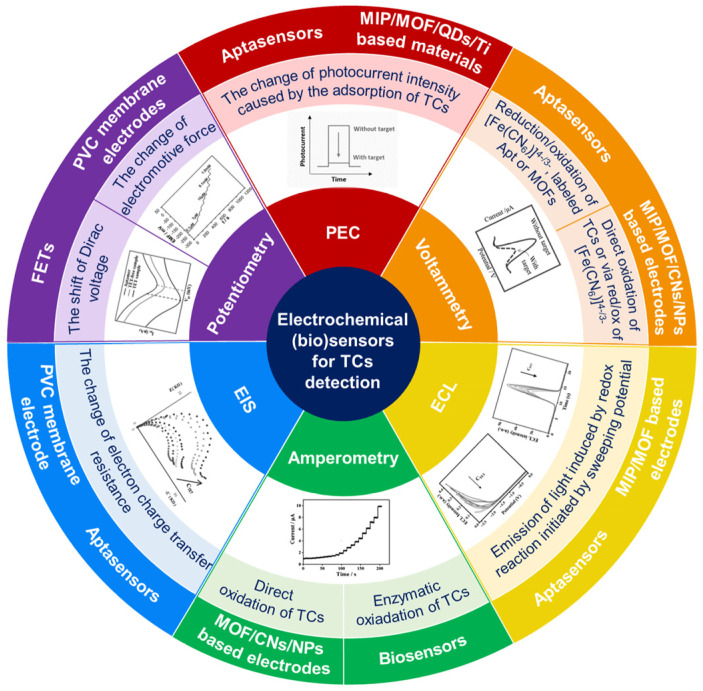
Recent sensing strategies of TCs using electrochemical (bio)sensors.

The sensors designed for TCs determination were often based on the functionalization of the electrode surface with molecularly imprinted polymers (MIPs) or metal–organic frameworks (MOFs). The purpose of utilizing these materials was to improve the analytical performance of the sensors produced. MIPs are artificial receptors that mimic the behavior of antibodies [23]. They are able to bind the analyte molecule in a similar way as an antigen binds to an antibody. The use of MIPs in the design of the sensors results in specificity and high sensitivity of the determination as well as the possibility of regenerating and reusing the sensor. Metal–organic frameworks, also known as porous coordination polymers, are crystalline compounds whose network structure is formed by coordination chemistry using metal cations and organic ligands [24]. MOF structure can be easily adapted to the desired goal by selecting appropriate metal cations and organic linkers. They are often employed in sensor development due to their unique characteristics, such as the presence of electroactive metal ions, high surface area, high porosity, tunable pore sizes, flexibility, and good thermal stability. Generally, MOFs are electrically insulating, therefore, the application of electron-conducting materials, such as carbon nanomaterials and metal nanoparticles, in the construction of electrochemical MOF-based sensors is necessary. In addition to the typical electrochemical sensors, with amperometric or potentiometric detection, a relatively large number of concepts of TCs quantification with photoelectrochemical detection have been proposed. The operating principle of the TCs (bio)sensors is summarized in Figure 3.

## 3. Recent Reports on Electrochemical (Bio)Sensors Designated for TCs Determination in Animal-Derived Foods

### 3.1. Milk Analysis

Zhang et al. [25] developed an aptasensor that allowed for the determination of tetracycline in milk samples using the differential pulse voltammetry (DPV) technique. To construct the aptasensor, the nanocomposite consisting of carbon nanofibers doped with iron and cobalt (Fe-Co@CNF) was utilized. This material was synthesized through the electrospinning technique followed by calcination. Then, the gold nanoparticles (AuNPs) were deposited onto the Fe-Co@CNF surface as a result of their in-situ synthesis through the reduction by ascorbic acid. The resulting nanocomposite was drop-casted on the surface of the glassy carbon electrode (GCE). The aptamer (5′-HS-(CH_2_)_6_-GAGAGACGGTGGTG-3′) was immobilized on the surface of Fe-Co@CNF@AuNPs/GCE by immersing the electrode in the aptamer solution for 2 h. Each component of the composite plays an important role in the sensor’s performance. The incorporation of Fe and Co improved the electrocatalytic performance, effective electrode surface area, and electron transfer efficiency of carbon nanofibers, while the presence of AuNPs significantly enhanced the electrical conductivity of the material and facilitated the effective immobilization of aptamer on surface of the composite through Au-S bonds. These characteristics contribute to the excellent electrochemical performance of the aptasensor. The operation of the sensor is based on the changes in the reduction peak current of the redox probe [Fe(CN)_6_]^4−/3−^, which occur due to TET–aptamer binding. As a result of tetracycline solution incubation on the surface of the aptasensor, TET molecules bind to the aptamer chains and block the electron transfer of [Fe(CN)_6_]^3−/4−^, which is reflected in the decrease in current response value. Such a drop in peak current is proportional to the concentration of TET. The aptasensor developed by Zhang et al. [25] is characterized by good analytical parameters, including high sensitivity (with a limit of detection (LOD) equal to 0.213 nM), a wide linear range, and good selectivity, even in the presence of other antibiotics, like ampicillin, kanamycin, metronidazole, or chloramphenicol. In order to verify the usefulness of the method, milk samples spiked with TET at three concentration levels (10–30 nM) were analyzed. The authors mention the need for preliminary preparation of milk samples for analysis, but do not specify the method of sample pretreatment. The aptasensor enabled the determination of TET with good accuracy, which was confirmed by recovery values ranging from 97.5% to 102.3% [25].

**Figure 4:**
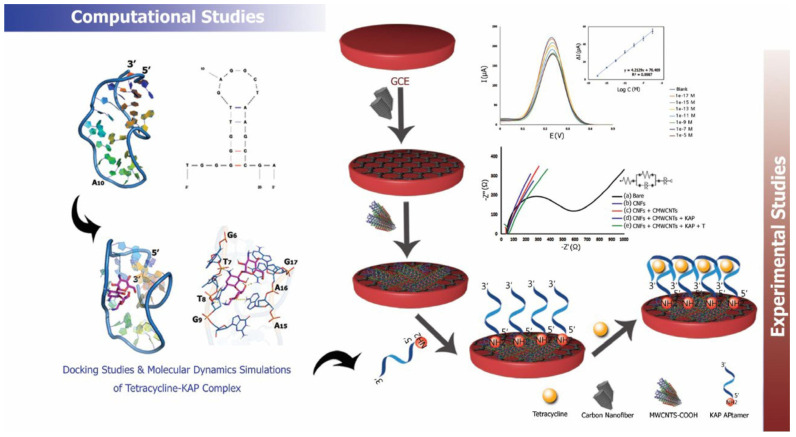
Schematic illustration of construction steps of the proposed aptasensor [26]. Reprinted from Ref. [26], copyright (2023), with permission from Elsevier.

A similar approach for the detection of TET in milk was described by Naseri and co-workers [26]. The GCE modified with CNFs and multi-walled carbon nanotubes functionalized with carboxylic groups (MWCNTs-COOH), deposited on the electrode surface by the layer-by-layer method, was used as the platform for aptamer immobilization. The design and construction steps of the aptasensor are depicted in Figure 4. In order to obtain the best electrochemical response, various aptamers, dedicated to the following antibiotics were considered: kanamycin (KAP), ampicillin (AMP), tetracycline (TET), and sulfadimethoxine (SUP). The interaction between antibiotics and the targeted sequences of aptamers, as well as the stability of antibiotic-aptamer complexes, was examined through molecular docking and molecular dynamic simulations. From these computational studies, the TET-KAP complex was identified as the most stable, and the KAP ssDNA (single-stranded DNA) with the 5′-NH_2_-C6-TGG GGG TTG AGG CTA AGC CGA-3′ sequence was used to fabricate the aptasensor for the highly sensitive detection of TET. The successful immobilization of KAP on the MWCNTs-COOH/CNFs/GCE surface was achieved via covalent bonding between activated carboxyl groups and amino groups at the aptamer strand’s ends. To block any remaining active sites and prevent non-specific adsorption, the aptasensor was incubated in a bovine serum albumin (BSA) solution for 1 h.

The BSA/KAP/MWCNTs-COOH/CNFs/GCE sensor was used in DPV measurements conducted in phosphate-buffered saline (PBS) pH 7.0 containing 5 mM of [Fe(CN)_6_]^3−/4−^. The linear correlation between the decrease in [Fe(CN)_6_]^3−/4−^ oxidation peak resulting from TET-KAP binding and the logarithm of TET concentration was used for antibiotic determination. The developed method showed excellent LOD (2.28 aM) and good selectivity in the presence of various drugs, such as kanamycin, ampicillin, or diclofenac. In order to perform milk analysis, it was necessary to subject the samples to preliminary preparation. To precipitate protein, fat, and other components, milk was sonicated with ethanol (mixed in a 1:2 volume ratio) and centrifuged. The milk serum was diluted with ultrapure water and spiked with TET (10^−12^–10^−6^ M). The obtained recovery values (96.45–104.18%) confirmed the excellent potential of the designed aptasensor for the determination of TET in real samples [26].

**Figure 5:**
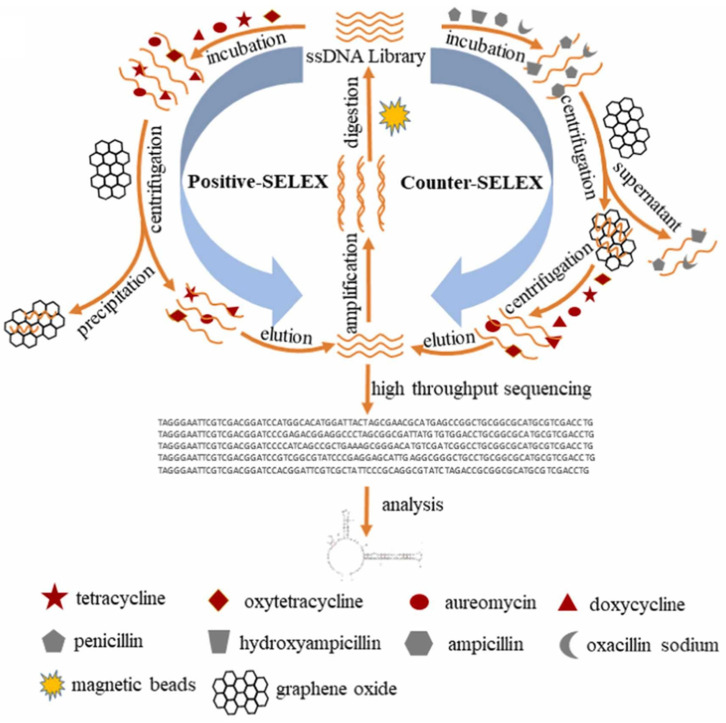
Schematic diagram of the GO-SELEX screening process for TCs [28]. Reprinted from Ref. [28], copyright (2024), with permission from Elsevier.

Guan et al. [27] and Bai et al. [28] developed aptasensors based on the same principle of operation. In the case of the sensor described by Guan et al., the broad-spectrum aptamer for TCs (5′-SH-(CH_2_)_6_-CGT ACG GAA TTC GCT AGC CCC CCG GCA GGC CAC GGC TTG GGT TGG TCC CAC TGC GCG TGG ATC CGA GCT CCA CGT G-3′) was immobilized on the surface of the GCE modified with the nanocomposite based on the combination of amino-functionalized flower-like zinc oxide nanoparticles, gold nanoparticles, and reduced graphene oxide. The aptasensor showed good sensitivity for TET, DOX, and OXY detection with a LOD equal to 0.33, 0.28, and 0.30 ng/mL, respectively. Furthermore, the interference study, involving seven different antibiotics, proved high selectivity of the aptasensor towards TCs. Such an aptasensor was successfully used for the determination of TET in spiked milk samples previously subjected to centrifugation and filtration [27]. Bai and co-workers selected a broad-spectrum aptamer for the determination of TCs based on the graphene oxide (GO)-SELEX screening (Figure 5) and molecular docking simulation to ensure the highest specificity and affinity [28]. On this basis, a voltammetric aptasensor was constructed by the modification of GCE with MWCNTs, Fe/Zn-modified montmorillonite (MMT), and aptamer using the layer-by-layer method. The developed aptasensor enabled a sensitive detection of four tetracyclines, TET, AUR, OXY, and DOX, with an LOD of 0.46 nM. The aptasensor was then employed for the simultaneous detection of TCs residues in milk. Spiking of the milk samples with a TCs solution was followed by the addition of trichloroacetic acid and centrifugation to eliminate the protein precipitate and the upper fat layer. 

Lastly, the samples were filtered using a sterile microfiltration membrane. The recovery rates of 98.26–104.15% demonstrated the potential of the aptasensor for sensitive analysis of TCs in food safety monitoring, offering a new approach for multi-residue antibiotic detection in milk [28]. 

Another approach for the determination of TET in milk is the application of biosensors based on labelled aptamers. Labeling the aptamer with compounds exhibiting redox properties, such as ferrocene [29,30] or methylene blue [31], eliminates the need to perform the voltammetric measurement in the presence of a model redox probe, as was described in the examples above. Malecka-Baturo et al. [29] prepared a biosensor by covalent co-immobilization of thiolated, ferrocene-labelled aptamer (5′–Fc–CCC CCG GCA GGC CAC GGC TTG GGTTGG TCC CAC TGC GCGT–(CH_2_)_3_–SH–3′) with 6-mercaptohexan-1-ol (MCH) onto a gold electrode (AuE) surface. Due to the presence of -SH groups in the aptamer chain, which show high affinity for Au, it was possible to immobilize the aptamer directly on the electrode surface. The use of MCH ensured the proper orientation of the aptamer and also blocked the gold electrode surface, thus eliminating the risk of direct TET oxidation without the participation of the biorecognition element. The analytical signal recorded by square-wave voltammetry (SWV) was based on changes in the redox behavior of ferrocene due to interactions between the aptamer and TET. The proposed aptasensor demonstrated a low LOD (0.16 nM) and high specificity even in the presence of other TCs, like OXY and DOX. The analysis of cow milk samples previously centrifuged and filtrated confirmed the practical potential of the Fc-Apt-MCH/AuE sensor for detecting TET in milk of animal origin [29].

A similar method was developed for the determination of OXY in milk samples using DPV. In this case, the aptasensor was created by immobilizing a ferrocene-labelled thiol-modified aptamer (5′-Fc-GGA-ATT-CGC-TAG-CAC-GTT-GACGCT-GGT-GCC-CGG-TTG-TGG-TGC-GAG-TGT-TGT-GTG-GAT-CCGAGC-TCC-ACG-TG-(CH_2_)_6_-SH-3′) onto the surface of a carbon screen-printed electrode (C-SPE) modified with gold nanostructures (AuNSs) of various geometries. By varying the concentration of HAuCl_4_, the type of supporting electrolyte, and the deposition technique (chronoamperometry, multipulse amperometry, and chronopotentiometry), nanostructures with fern-like, spear-like, cauliflower-like, thistle-like, and tetragonal pyramid shapes were obtained. The aptasensor constructed using the C-SPE with deposited thistle-like AuNSs exhibited the best performance, offering the highest ferrocene signal and the largest effective area. Interestingly, Blidar et al. used pulse-assisted deposition for the aptamer immobilization, which allowed them to shorten this stage of sensor preparation from 2 h to a few minutes. Similarly to the previous work, for the blocking step, MCH was used. The aptasensor showed acceptable sensitivity towards OXY detection, with LOD equal to 8.7 nM and good selectivity in the presence of various antibiotics, such as TET, amoxicillin, ampicillin, gentamicin, and vancomycin. It is worth mentioning that the authors investigated the regenerability of the aptasensor. After three series of measurements, the current response of the aptasensor retained ca. 93% of its original response value, which confirmed the possibility of reusing the aptasensor multiple times. For the sample analysis, cow milk was spiked with OXY. To precipitate proteins and fats, HClO_4_ was added to samples, which were subsequently centrifuged, and then the antibiotic was determined in the collected supernatant. The aptasensor enabled determination of OXY with good accuracy, which confirmed its usefulness for real sample analysis [30].

**Figure 6:**
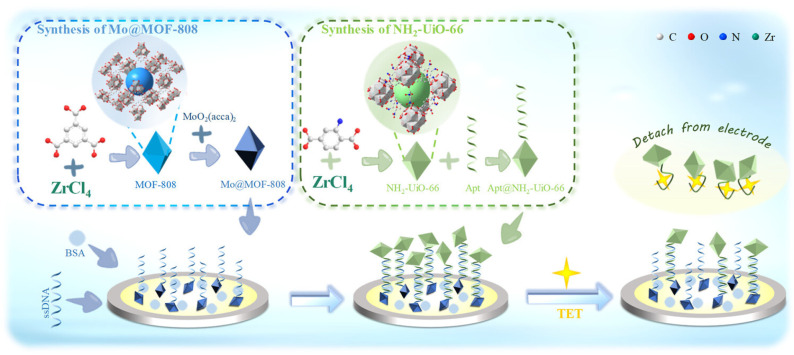
Principle of the ratiometric electrochemical biosensor for TET detection based on Mo@MOF-808 and NH_2_-UiO-66 [32]. Reprinted with permission from [32]. Copyright 2023 American Chemical Society.

Liang et al. [32] presented an innovative biosensor with a dual-signal response based on electrochemically active metal−organic frameworks of Mo@MOF-808 and NH_2_-UiO-66 for ultrasensitive detection of TET in milk. The biosensor was prepared by the GCE modification with AuNPs, Mo@MOF-808, activated ssDNA, and BSA using the layer-by-layer method. The effective immobilization of the DNA strand on the sensor surface was possible due to the presence of a carboxyl group at the 5′ end of ssDNA and −NH_2_ on the surface of Mo@MOF-808. In parallel, the complementary aptamer was incubated with NH_2_-UiO-66, and the obtained material was incubated with TET for 2 h. Finally, BSA/ssDNA/Mo@MOF-808/AuNPs/GCE was incubated with TET/Apt@NH_2_-UiO-66 for 40 min and washed with ultrapure water. The schematic representation of the procedure of biosensor preparation is shown in Figure 6.

In DPV measurements, the biosensor displayed two peaks corresponding to the oxidation of NH_2_-UiO-66 and the reduction of Mo@MOF-808. In the presence of TET, the aptamer hybridized, causing the double strand to melt and leading to the detachment of Apt@NH_2_-UiO-66 from GCE. It resulted in a decrease in the NH_2_-UiO-66 signal and an increase in the Mo@MOF-808 signal. Thus, the proposed sensor enabled the detection of TET through the simultaneous change of two electrochemical signals. Based on these signals, the anodic-to-cathodic peak ratio was calculated. This analytical signal demonstrated a great linear correlation over the wide concentration range with outstanding sensitivity (LOD of 0.009792 nM). The constructed sensor was successfully applied to detect TET in milk samples (subjected to preliminary preparation in a similar manner to that described in [26]), suggesting excellent application prospects [32].

An interesting application of a biosensor for the determination of TET in milk is the paper presented by Beilison et al. [33]. The proposed biosensors were modified using various nanomaterials, like reduced graphene oxide (rGO), multi-walled carbon nanotubes (MWCNTs), gold nanoparticles (AuNPs), and chitosan, along with an immobilized tyrosinase (TYR) enzyme. In this paper, four types of sensors based on screen-printed electrodes (SPEs) were presented, but the surface of each electrode was modified in a different way: SPE/Chitosan/MWCNTs/TYR; SPE/Chitosan/MWCNTs/AuNPs/TYR; SPE/Chitosan/RGO/TYR, and SPE/Chitosan/RGO/AuNPs/TYR. The electroanalytical assay was based on measuring the inhibition of the tyrosinase enzyme by tetracycline. When tetracycline was present, it interfered with the enzyme’s ability to convert phenol to quinone, leading to a decrease in the signal (current). This decrease in signal was proportional to the concentration of tetracycline in the sample. For each of the proposed sensor versions, the linearity range was determined (generally 1 nM–10 μM); however, the lowest detection limit (50 pM) was obtained for the SPE/Chitosan/MWCNTs/AuNPs/TYR modification. The milk sample (1.5% fat) was diluted 50 times with a phosphate-buffer solution to reduce the influence of the matrix components. The detection limit given by the authors is below the TET content in milk required by the standards.

An example of a DPV sensor dedicated to the determination of tetracycline in milk is MSMIP/rGO/GCE [34]. This molecularly imprinted electrochemical sensor for TET detection was obtained by dropping the reduced graphene oxide (rGO) and magnetic surface molecularly imprinted polymer (MSMIP) as supports directly onto a glassy carbon electrode in order to enhance the conductivity and selectivity of the sensor. The preparation of the magnetic surface-imprinted polymer Fe_3_O_4_@mTiO_2_@MIP (MSMIP) included several synthesis steps, including the preparation of Fe_3_O_4_ nanoparticles, Fe_3_O_4_@SiO_2_ microspheres, Fe_3_O_4_@SiO_2_@mTiO_2_ microspheres, and finally MSMIP nanoparticles prepared by surface-initiated RAFT polymerization. The entire procedure of preparing the mixture for the modification of the sensor surface required several days, high temperatures (even 400 °C), numerous reagents, including organic solvents, and finally 48 h extraction in a Soxhlet apparatus. To evaluate the analytical usefulness of the proposed sensor, DPV was used. The linear response to the tetracycline oxidation reaction, under optimized conditions, was observed in the concentration range of 1.6 nM to 88 nM with a detection limit of 0.916 nM. The preparation of the milk sample for analysis was a bit more demanding and consisted of mixing the sample with anhydrous ethanol. After waiting for 20 min, the mixture was filtered and then diluted with phosphate-buffer solution. The obtained results showed that the accuracy expressed as recovery rate was between 97.20% and 101.50% with a precision expressed as relative standard deviation smaller than 5.0%.

**Figure 7:**
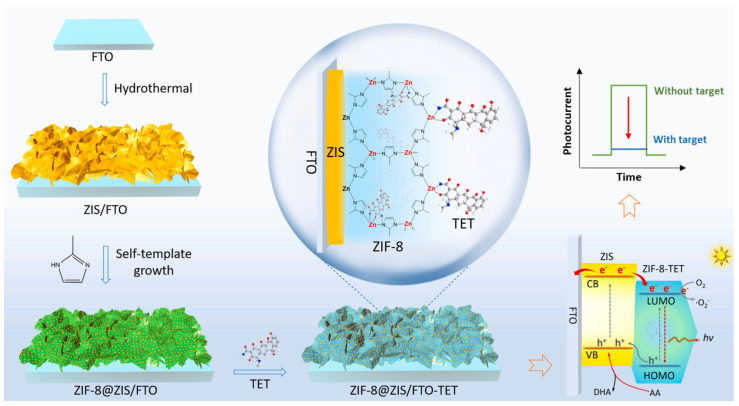
The scheme of ZIF-8@ZIS/FTO synthesis for photoelectrochemical TET detection [35]. Reprinted from Ref. [35], copyright (2022), with permission from Elsevier.

For the analysis of tetracycline-spiked milk and river water samples, Chen et al. proposed the use of MOFs in combination with photoelectrochemical (PEC) detection [35]. The sensor design was based on the use of a fluorine-doped tin oxide (FTO) electrode, on which a semiconductor structure of ZnIn_2_S_4_ (ZIS) nanosheets was synthesized using a hydrothermal method (Figure 7). The use of ZIS is related to its properties that are particularly desirable in the construction of PEC-type sensors: stability, narrow bandgap, visible light response ability, proper conduction and valence band position, and direct transport pathway for photogenerated carriers. Zeolitic imidazolate framework-8 (ZIF-8) was then spontaneously deposited on the ZIS surface and used as a recognition element in the sensor. It was experimentally verified that neither Zn(II) nor tetracycline solutions alone generated fluorescence. Obvious fluorescence was observed at 517 nm for the mixture of both solutions. In the presence of TET, only the ZIF-8-based sensor could generate strong fluorescence, suggesting a strong interaction between tetracycline and ZIF-8. The final ZIF-8-TET complex could be excited, while the photogenerated electrons on the conduction band of ZIS would partly transfer into the lowest unoccupied molecular orbital (LUMO) of ZIF-8-TET instead of FTO, thus leading to the quenched photocurrent signal. With subsequent additions of tetracycline, the analytical signals decreased in a very wide range from 1 pM to 700 μM. The decrease in fluorescence intensity was proportional to the logarithm of TET concentrations in a linear range from 1 pM to 100 nM, with good linearity (R^2^ = 0.995) and a very low detection limit of 0.1 pM. The recoveries obtained for tested real samples (river water and milk spiked at three concentration levels) ranged from 97% to 108%. The influence of potential interferents, including bovine serum albumin and popular inorganic cations and anions, as well as the drugs kanamycin, oxytetracycline, hygromycin, puromycin, and ofloxacin, was also examined. It was proven that only TET can cause a significant reduction in the signal, which confirms the good selectivity of the sensor.

### 3.2. Honey

An unusual approach to the analysis of honey (as a model fuel for microbial fuel cells, MFC) and water samples was presented by Massaglia et al. [36]. The general principle of MFC is based on the presence of electroactive biofilms (as biocatalysts) that convert chemical energy from organic matter into electrical energy. In the presence of contaminants that act as biofilm toxicants, such energy conversion can be significantly reduced. In the paper, the effectiveness of MFCs as a tool to detect the presence of antibiotics (TET) in small amounts in honey was checked. Honey was selected as the sample for the study, for which the maximum allowable residue limit (MRL) of tetracycline for human consumption has been set at 20 µg/kg by the European Union. The second reason for choosing this matrix (beyond the analytical aspects) is its application as a carbon source for anodic microorganisms in MFCs. The measurement system used in the tests consisted of two electrodes. Anode was based on the carbon paper (CP) decorated with nanofibers (NFs) made of polyethylene oxide (PEO)—CP/PEO-NFs. Commercial CP electrodes decorated with polytetrafluoroethylene (PTFE) on the outer side (the one in contact with air) and by a layer of Pt/C-based catalyst on the inner side (in contact with the electrolyte) were used for cathode preparation. The principle of the assay was to use the ability of electroactive biofilms to adapt their metabolism, that is, to detect the presence of small amounts of tetracycline due to the strong influence that TET has on the metabolic activity of cells, but also to generate electrical energy in an MFC-type device to power itself. The biofilm (as the sensing element) metabolism was directly responsible for the conversion of the chemical signal associated with the fuel and analytes available in the electrolyte into the output electrical signal (current density). The effect of tetracycline in the concentration of 3.53 µg/kg (6 times lower than the MRL) on the metabolic activity of microorganisms was confirmed.

In order to obtain a low detection limit (3.3 fM), Li et al. proposed for TET determination to combine an aptasensor with photoelectrochemical detection [37]. The functioning of the sensor was verified in the analysis of various food products, including honey. The sensor design comprised the preparation of a 3D ZnO and AuNPs mixture and its deposition on the ITO (indium tin oxide) surface. MoS_2_ nanosheets and the aptamer of TET were mixed to form MoS_2_/ZnO/Au/ITO with the immobilized TET aptamer by self-assembly. The task of the aptamer (5′-CGTAC GGAAT TCGCT AGCCC CCCGG CAGGC CACGG CTTGG GTTGG TCCCA CTGCG CGTGG ATCCG AGCTC CACGT G-3′) was to specifically recognize the analyte molecules, while the presence of MoS_2_ allowed for the adsorption of TET aptamers via π–π interactions and the formation of a 3D ZnO/Au heterostructure by self-assembly in solution. TET is easily captured by the aptamer on the MoS_2_ surface. This process promotes the separation of photogenerated electron–hole pairs in the MoS_2_/ZnO/Au heterostructure and thus improves the photocurrent signal response of the system by consuming the photogenerated holes. Two TET-oxidation pathways are proposed: direct oxidation reaction of photogenerated holes in the MoS_2_ valence band to TET and indirect oxidation of TET by •OH. Hydroxyl radicals are generated in the oxidation reaction between photogenerated holes and OH^−^ or H_2_O. Both pathways are assumed to consume the photogenerated holes in the MoS_2_ valence band, which will reduce the charge recombination efficiency, increase the photocurrent response, and ultimately serve the purpose of TET detection. The analytical utility was checked for four spiked food samples, in addition to honey, in milk, pig kidneys, and pork. Their preparation method included dilution with phosphate-buffer solution and adding trichloromethane and trichloroacetic acid for each of the samples. Then, the samples (milk, honey, and meat slurry) were sonicated to completely dissolve them and centrifuged to ensure complete removal of proteins and other organic matter. The supernatant was collected and centrifuged again to remove the sediment. TET was added to the prepared samples at concentrations of 10 and 100 pM. The recovery ranged from 94.2% to 104.45%, with relative standard deviations (RSD) below 5% (*n* = 3). This confirms the wide application possibilities of the constructed sensor.

### 3.3. Other Food Products

Two works presented below attempted to determine tetracycline in more demanding matrices, such as chicken meat. Also, both papers present results based on measurements using the DPV technique. According to Yang et al. [38], for the first time, the combination of surface molecularly imprinted magnetic covalent organic frameworks (Fe_3_O_4_@COFs@MIPs) with a disposable screen-printed electrode (SPE) allowed them to construct a portable and on-site electrochemical sensor for rapid tetracycline detection in spiked chicken meat, water, and milk. The construction of the proposed sensor can be achieved in three steps. In the first step, magnetic covalent organic frameworks (COFs) were synthesized as a support material for the MIPs (molecularly imprinted polymers). Subsequently, MIPs were polymerized on the surface of the magnetic COFs to form Fe_3_O_4_@COFs@MIPs, which provided specific recognition sites for TET molecules. In the final step, the Fe_3_O_4_@COFs@MIPs were deposited on a surface of SPE to simplify the electrode-modification process and enable the development of an electrochemical sensor for sensitive and rapid TET detection. The preparation of chopped chicken as well as milk samples required the following procedure: chopped chicken samples (with a 10% trichloroacetic acid aqueous solution and chloroform) were vortex mixed to precipitate organic molecules such as proteins and lipids in the matrix. After being centrifuged, the supernatant was filtered through a nylon filter membrane for sensing detection by the proposed magnetic molecularly imprinted electrochemical sensors (MMIECSs). Under optimal conditions, linearity in the range from 1 × 10^−10^ to 1 × 10^−4^ g/mL was achieved. The recovery for all actual samples was between 96.15% and 106.20%, and the RSD did not exceed 2.5%.

**Figure 8:**
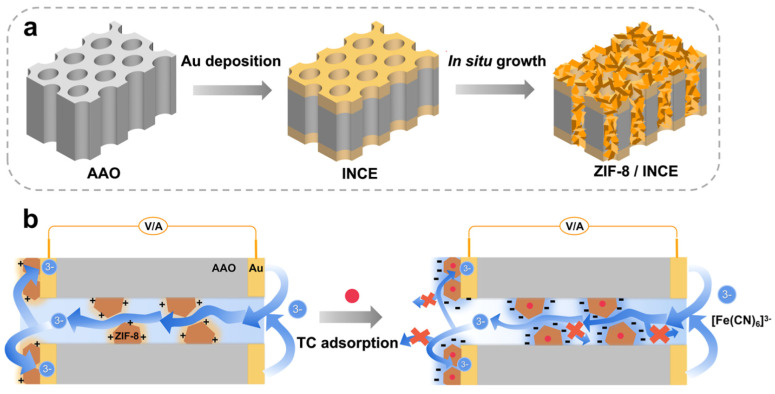
Modification of inner and outer surface of the INCE; (**a**) schematic of the fabrication process of the ZIF-8/INCE nanochannel sensor, (**b**) illustration of the response process of ZIF-8/INCE to tetracycline: depicting the ion transportation in nanochannels and on the outer surface in the presence of a nanoconfined environment [39]. Reprinted with permission from [39]. Copyright 2023 American Chemical Society.

As a new model of nanoelectrochemistry, as well as for providing an alternative solution in the field of nanopore analysis for small molecules such as tetracycline, the fabrication of an integrated nanochannel electrode (INCE) was proposed [39]. To build INCE, an anodic aluminum oxide (AAO) nanochannel chip was deposited in nanoporous gold layers both in the inner channel and on the outer surface (Figure 8). Two-sided modification by MOFs (due to their adsorption properties) allowed the pore size to be reduced to a few nanometers, which is within the range of the electrical double-layer thickness for limited ion diffusion. For this reason, zeolite imidazole framework-8 (ZIF-8), known for its structural stability and selective adsorption of small molecules, was employed. Additionally, for sensor miniaturization, the gold electrode integrated into the nanochannels of the INCE facilitated redox reaction probing. Ionic current measurement, connected with the reduction process of the [Fe(CN)_6_]^3−^, has been the dominant signaling strategy in nanochannel-based sensors. To evaluate the feasibility of ZIF-8/INCE for practical application, the authors used the sensor to detect TET in purchased chicken samples. Fresh chicken breast samples were homogenized and mixed with an organic solvent: ethyl acetate. After ultrasonication and centrifugation of the mixture, the supernatant was collected and diluted 10 times with ultrapure water for TET detection by the standard addition method. Finally, results showed that the recovery rate ranged from 81.28% to 97.19%, with an accuracy at an acceptable level for natural samples. These results suggested that the proposed strategy is promising in practical applications. Moreover, the use of the external surface as an electrode eliminated the need for additional electrodes, which was a key aspect in developing a micro-/nanoelectrochemical system and a miniaturized device.

The nanomaterials fabricated using electrospinning technology were also used for the construction of biosensors intended for TET detection in food products such as chicken ham [40]. The authors prepared a carbon nanofiber (CNF) electrode from polyacrylonitrile by the electrospinning process followed by carbonization at 1000 °C. The CNFs were spherically perforated (5 mm) and attached to the copper wire for electrical contact. Gold nanoparticles were electrochemically deposited on the surface of the CNF electrode via chronoamperometry. Then, the thiolated aptamer (5′-HS-(CH_2_)_6_-CGT ACG GAA TTC GCT AGC CCC CCG GCA GGC CAC GGC TTG GGT TGG TCC CAC TGC GCG TGG ATC CGA GCT CCA CGTG-3′) was immobilized onto its surface via Au-S bonds. To exclude non-specific adsorption of TET, the aptasensor surface was blocked with BSA. After incubation with TET solution, the biosensor was used in cyclic voltammetric (CV) measurements carried out in KCl solution containing [Fe CN)_6_]^3−/4−^ as a redox probe. The operation of the sensor was based on the proportional decrease in the peak current associated with the hindering of electron transfer between hexacyanoferrate ions and the aptasensor surface as a result of TET-aptamer binding. The biosensor showed a satisfactory LOD (0.12 nM) and high reproducibility and repeatability (RSD < 3%). For sample analysis, the chicken ham was comminuted in a commercial blender. Then, the resulting suspension was diluted and spiked with different concentrations of antibiotics. The developed aptasensor enabled accurate determination of TET in the presence of the meat matrix (with recoveries of 96.92–104.31%), which confirms its usefulness in food control [40].

**Figure 9:**
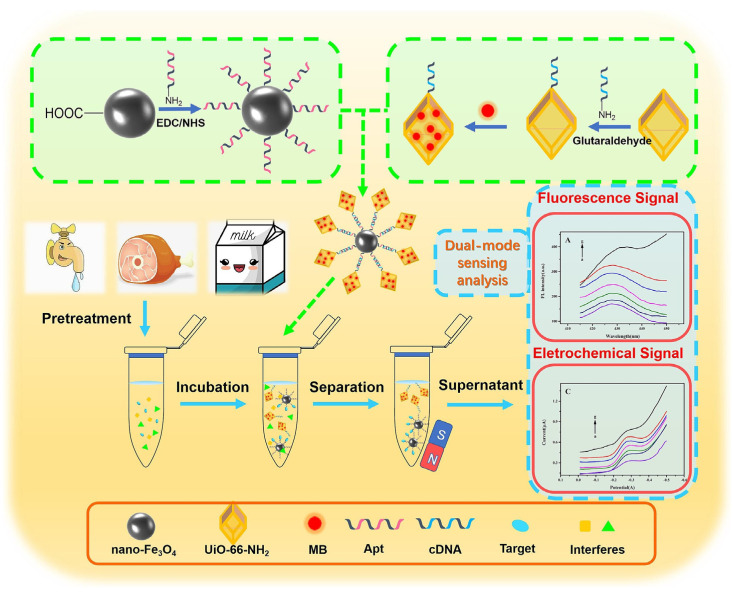
Schematic illustration of biosensing platform construction and the dual-signal mode biosensing strategy [41]. Reprinted from Ref. [41], copyright (2024), with permission from Elsevier.

An interesting approach to the determination of TET using dual fluorescent/electrochemical detection was proposed by Yang et al. [41]. The sensing platform was designed based on an aptamer with a high affinity towards TET (5′-NH_2_-CGT ACG GAA TTC GCT AGC CCC CCG GCA GGC CAC GGC TTG GGT TGG TCC CAC TGC GCG TGG ATC CGA GCT CCA CGT G-3′), selected by means of the computer simulation. The sensor was constructed using Apt-functionalized nano-magnetic beads (Fe_3_O_4_-Apt) as a capture probe and Apt-complementary short-chain functionalized fluorescent MOF (UiO-66-NH_2_) loaded with methylene blue (cDNA-MOF-MB), involving complementary pairing of Apt and cDNA. When the sensing platform was contacted with the TET solution, a target-induced structural conversion—the dissociation of the complementary pair of Apt and cDNA—occurred, resulting from a preference for binding the Apt to the target (TET) rather than to cDNA-MOF-MB. Simultaneously, corresponding dual-signal tags into the supernatant were released. After magnetic separation, the supernatant was used for the fluorescent/electrochemical assay. The higher the TET concentration, the more signal tags were released, and the fluorescence intensity of the MOF and MB signal current also increased. The stages of the sensor’s construction and a scheme of its operation can be seen in Figure 9. The labelled biosensing platform (Fe_3_O_4_-Apt/cDNA-MOF-MB) exhibited low detection limits, equal to 1.69 × 10^−10^ g/mL and 1.15 × 10^−10^ g/mL for fluorescence and electrochemical-mode assays, respectively. Its selectivity of operation in the presence of DOXY, OTC, and AUR and usefulness in analyses of spiked food samples, such as chicken, milk, and tap water, were confirmed. For analysis, chicken meat was minced, diluted with water, vortexed with chloroform and 10% trichloroacetic acid, and centrifuged twice. The milk sample was subjected to the same preparation (without mincing). The tap water sample was filtered through a 0.22 μM nylon filter. The recovery values were 95.01–101.30% and 94.99–100.63% for fluorescence and electrochemical detections, respectively, and were consistent with the results of HPLC determination.

The remaining examples of electrochemical (bio)sensors used for the detection of TCs in food products are summarized in Table 1.

**Table 1:** Electrochemical (bio)sensors used for the detection of tetracyclines in food products.

Sample	Type of Sensor	Analyte	Electrode Configuration	Electroanalytical Assay	Detection Method	LOD	Linear Range	Ref.
Milk	Aptasensor	TET	Fe-Co@ CNF@AuNPs@Apt/GCE	Reduction in [Fe(CN)_6_]^4−/3−^	DPV	0.21 nM	5.12 nM–10 mM	[25]
Milk	Aptasensor	TET	BSA/KAP/MWCNTs-COOH/CNFs/GCE	Oxidation of [Fe(CN)_6_]^4−/3−^	DPV	2.28 aM	10 aM–10 µM	[26]
Milk	Aptasensor	TET DOX OXY	rGO-ZnO-AuNPs/Apt/BSA/GCE	Oxidation of [Fe(CN)_6_]^4−/3−^	CV	0.74 nM 0.61 nM 0.68 nM	2.25 nM–2.25 µM	[27]
Milk	Aptasensor	TET AUR OXY DOX	Apt-Fe/Zn-NaMMT-MWCNTs/GCE	Reduction in [Fe(CN)_6_]^4−/3−^	DPV	0.46 nM	1 nM–10 µM	[28]
Milk	Aptasensor	TET	Fc-Apt-MCH/AuE	Oxidation of ferrocene	SWV	0.16 nM	0.1–1.0 nM	[29]
Milk	Aptasensor	OXY	Fc-Apt-MCH/AuNSs/ C-SPE	Oxidation of ferrocene	DPV	8.7 nM	0.05–1.2 µM	[30]
Milk	Aptasensor	TET	BSA/ssDNA/Mo@MOF-808/AuNPs/GCE	Dual response: reduction in Mo@MOF-808 and oxidation of NH_2_-UiO-66	DPV	9.79 pM	0.1 nM–10 µM	[32]
Milk	Voltammetric	TET	MSMIP/rGO/GCE	Oxidation of TET	DPV	0.92 nM	1.6–88 nM	[34]
Milk	Voltammetric	TET	AuNSs/AuE	Direct detection of TET-Fe(III) complex	DPV	345 nM	0.1–100 μM	[42]
Milk	ISE	AUR DOX OXY TET	PVC-CB [8]-FNDPE-TCPB-MWCNTs/CPE	Change in electromotive force	HPLC-ISE	0.03 µM (AUR, DOX, TET) 0.1 µM (OXY)	0.1–10 µM	[43]
Milk	Photoelectrochemical	TET	Ov-TNTs/Ti	Decrease in photocurrent with TET concentration	PEC	0.33 nM	0.1–1000 nM	[44]
Honey	ISE	TET	([o-COSAN]^−^)-TC/ PVC/SWCNT/AuME	The change in electron charge transfer resistance	EIS	0.75 fM	2.25 fM–11.2 pM	[45]
Milk, honey, pig kidneys, pork	Aptasensor	TET	Apt/MoS_2_/ZnO/Au/ITO	Increase in photocurrent intensity with TET concentration	PEC	3.3 fM	10 fM–10 nM	[37]
Chicken	Voltammetric	TET	ZIF-8/INCE Nanochannel Sensor	Reduction in [Fe(CN)_6_]^3−^	DPV	0.23 µM	0.9–2 250 µM	[39]
Chicken ham	Aptasensor	TET	Apt/Au/CNF	Oxidation of [Fe(CN)_6_]^4−/3−^	CV	0.12 nM	1.0–100 µM	[40]
Milk, meat	Voltammetric	TET	CO_2_-LIG-MIP/PI	Decrease in current intensity due to filling of MIP gaps by TET	DPV	0.8 nM	10–300 nM	[46]
Milk, chicken feedstuff	Voltammetric	TET	MIP/GO/Al_2_O_3_/AuE	Oxidation of [Fe(CN)_6_]^4−/3−^	DPV	0.17 nM	0–60 nM	[47]

Detection method: DPV—differential pulse voltammetry, CV—cyclic voltammetry, SWV—square-wave voltammetry, HPLC-ISE—high-performance liquid chromatography—ion-selective electrode, PEC—photoelectrochemistry, EIS—electrochemical impedance spectroscopy. Electrode configuration: CNF—carbon nanofiber, AuNPs—gold nanoparticles, Apt—aptamer, GCE—glassy carbon electrode, BSA—bovine serum albumin, KAP—kanamycin, MWCNTs-COOH—(-COOH) functionalized multi-walled carbon nanotubes, rGO—reduced graphene oxide, MMT—montmorillonite, MCH—6-mercaptohexan-1-ol, AuE—gold electrode, AuNSs—gold nanostructures, C-SPE—carbon screen-printed electrode, ssDNA—single-stranded DNA, MOF—metal–organic framework, MSMIP—magnetic surface molecularly imprinted polymer, PVC—polyvinyl chloride, CB[8]—cucurbit[8]uril hydrate, FNDPE—2-fluorophenyl 2-nitrophenyl ether, TCPB—potassium tetrakis(p-chlorophenyl)borate, Ov-TNTs—oxygen vacancy-regulated TiO_2_ nanotube arrays, SWCNT—single-walled carbon nanotube, ITO—indium tin oxide, ZIF-8—zeolite imidazole framework-8, INCE—integrated nanochannel electrode, CO_2_-LIG—CO_2_ laser-induced graphene, MIP—molecularly imprinted polymer, PI—polyimide.

## 4. Recent Reports on Electrochemical (Bio)Sensors Designated for TCs Determination in Environmental Samples

### 4.1. Water

Song et al. [48] described the voltammetric method of TET determination in various samples, including tap water. The aptasensor construction was based on the GCE covered with carbon nanotubes modified with tungsten trioxide (WO_3_@CNTs) and gold nanoparticles. The WO_3_@CNTs@AuNPs was used as a scaffold for the immobilization of 5′-thiolated TC aptamer (5′-SH-CGT ACG GAA TTC GCT AGC CCC CCG GCA GGC CAC GGC TTG GGT TGG TCC CAC TGC GCG TGG ATC CGA GCT CCA CGT G-3′). The modification of CNTs with WO_3_ ensured efficient electron transfer, resulting in an amplification of the electrical signal. The combination of WO_3_@CNTs and AuNPs resulted in mutual reinforcement of the electrochemical properties of individual nanocomponents, the so-called synergistic effect, which additionally improved the sensor response and facilitated the effective bonding of the aptamer on the sensor surface via Au-S bonds. To prevent non-specific adsorption, BSA was used to block the remaining sites on the electrode’s surface. For TET detection, the aptasensor was incubated with the antibiotic solution, washed, and then DPV measurements were performed using [Fe(CN)_6_]^3−/4−^ as a redox probe. The operation of the sensor is analogous to the previously described examples [24,25]. The aptasensor exhibits good analytical parameters, including high sensitivity (LOD of 0.048 nM) and good selectivity in the presence of other antibiotics. The usefulness of the method was verified through the determination of TET in various water and food samples, i.e., tap water, pond water, and river water, as well as milk, honey, and black tea infusion. Prior to the analysis, samples were sonicated and centrifuged, the supernatant was carefully aspirated, and finally, spiked with TET. The analysis of samples demonstrated acceptable accuracy of the aptasensor, with recoveries ranging from 91.3% to 114.4% [48].

A perovskite quantum dot-based ECL sensing strategy of OXY in tap water was developed by Chen et al. [49]. In order to ensure luminescent properties, perovskite nanocrystals (PNCs), synthesized by the hot injection method in the presence of cetyltrimethylammonium bromide (CTAB-PNC), were used for GCE modification. The carboxyl- modified capture ssDNA (Apt1-com) was immobilized on the surface of CTAC-PNC/GCE, followed by aptamer (Apt1), which bound to the capture ssDNA and resulted in the formation of double-stranded DNA (dsDNA). Independently, the thiol-modified competing chain of ssDNA (Apt1-anti), labeled with Ag nanoclusters (AgNCs), was incubated with OXY and then with BSA. Finally, Apt1-anti-Ag-BSA was incubated with dsDNA/CTAB-PNC/GCE. The use of perovskite NCs provided a strong and stable initial electrochemiluminescence signal, while the catalytic activity of AgNCs enhanced the recognition sensitivity of the biosensor. The ECL measurements were conducted in the PBS solution containing potassium persulfate. The principle of sensor operation is based on the basic ECL-emission mechanism described in Section 2. The described ECL-sensing strategy is characterized by an LOD equal to 27 nM and good selectivity. The biosensor was successfully used for the determination of OXY in spiked tap water samples, as well as the differentiation of three TCs, OXY, TET, and DOX, with the use of the principal component analysis (PCA) method. The obtained recoveries (94.7–101.6%) confirmed the practical value of this method for the determination of OXY in real samples, while PCA results proved the good discrimination ability of the developed ECL system [49].

The synergistic effect of the well-known electrode surface modifiers multi-walled carbon nanotubes (MWCNTs) and gold nanoparticles (AuNPs), in combination with the mentioned above metal–organic frameworks (MOFs), was presented in the paper by Weng et al. [50]. Preparation of the sensor for measurements consisted of a few simple steps: firstly, application of a mixture of MOF (Zr-UiO-66) and MWCNTs on the surface of GCE and then electrodeposition of gold nanoparticles. This design of the amperometric sensor allows for its quick and easy regeneration, similar to aptasensor regeneration. The analytical signal is the current related to TET oxidation. The wide linearity range, from 0.5 µmol/L to 0.225 mmol/L, as well as its selectivity and the stability of the sensor operation, allowed its use in the determination of tetracycline in spiked lake water samples. In two cases, recovery close to 100% was obtained with a standard deviation of results below 3.5%. As a conclusion, it can be stated that the proposed sensor provides a promising and prospective MOFs-based sensing platform for the detection of tetracyclines in simple real samples (water).

An interesting example of an ultrasensitive ECL sensor for TET determination in river water samples was described by Han et al. [51]. The integration of silver nanoparticles (AgNPs) with the porphyrin metal−organic matrix (Ag@AgPOM) constituted the basis of a bifunctional ECL system. Such a composite exhibited excellent electrocatalytic performance and acted as both the ECL emitter and co-reaction accelerator. For the biosensor preparation, the GCE was modified with Ag@AgPOM, and then the capture strand (cDNA) was immobilized onto its surface while MCH was used to block the nonspecific sites. In order to detect TET, the MCH/cDNA/Ag@AgPOM/GCE was incubated with an antibiotic solution, and the ECL response of the biosensor was measured in PBS solution (pH 7.4) containing S_2_O_8_^2−^ ions. The sensor operation was based on the basic ECL mechanism (described in Section 2). The developed biosensor showed a wide linear-detection range towards TET, with a low detection limit of 0.14 fM. Prior to the analysis, natural river water samples were filtered through a microporous membrane. Then, ethyl acetate and sodium chloride were added to samples and mixed using a vortex. This mixture was centrifuged, and the obtained supernatant was dried under nitrogen. Finally, the solid residue was dissolved in ultrapure water. A similar procedure was used for milk sample preparation. The recovery rates obtained for spiked river water and milk samples ranged from 93.6% to 109%, which confirmed that the developed method can be successfully used to monitor TET residues in environmental samples [51].

A novel, covalent organic frameworks-based electrochemical/fluorescence dual-mode aptasensor for TET detection in river water, among others, was proposed by Ling et al. [52]. To fabricate the sensor, aptamers for TET (5′-SH-(CH2)_6_-GTT TGT GTA TTA CAG TTA TGT TAC CCT CAT TTT TCT GAA C-3′) were immobilized onto the glassy carbon electrode (GCE) previously modified with molybdenum disulfide nanosheets (MoS_2_ NSs), exhibiting excellent electrochemical properties, and gold nanoparticles (AuNPs). The fluorescent covalent organic frameworks (COFs) were modified, in their pores, with electrochemically active methylene blue (MB), gold nanoparticles, and, by self-assembly, single-stranded DNA (ssDNA) complementary to the APT chain. When contacting the surface of Apt-AuNPs@MoS_2_ NS-GCE with ssDNA-MB@AuNPs@COF, complementary pairing of Apt and ssDNA took place. In the presence of target TET, it was recognized by Apt and induced the removal of ssDNA-MB@AuNPs@COF from the electrode surface. This resulted in an enhancement of the fluorescence intensity and in the lowering of the electrochemical signal. Selectivity tests performed for other antibiotics (chlorotetracycline, oxytetracycline, penicillin, kanamycin, erythromycin, streptomycin, chloramphenicol), metal cations (Ca, Na, Mg, Al, Fe), amino acids (cysteine, lysine, aspartic acid, serine, histidine, phenylalanine), and carbohydrates (glucose, lactose, galactose) as potential interferences confirmed the good performance of the sensor in this regard. The proposed sensor was characterized by low limits of detection: 0.03 nM for the electrochemical mode and 0.05 nM for the fluorescent detection. Its functioning was verified in analyses of spiked river water samples and milk samples. River waters were centrifuged to remove large particles and filtrated using 0.22 µm PTFE membranes to eliminate the suspended matter. Then, they were spiked with TET solutions of different concentrations and taken for analysis. Milk samples were spiked with TET solutions, then mixed with chloroform and trichloroacetic acid, sonicated to precipitate proteins and fattiness, and centrifuged. The supernatant was filtered through a 0.22 μm polytetrafluoroethylene (PTFE) membrane, and the filtrate was analyzed. Satisfactory recovery values were obtained, 91.2–106.8% for electrochemical detection mode and 91.2–106.8% for fluorescent one, showing good accuracy of the proposed dual-mode aptasensor.

**Figure 10:**
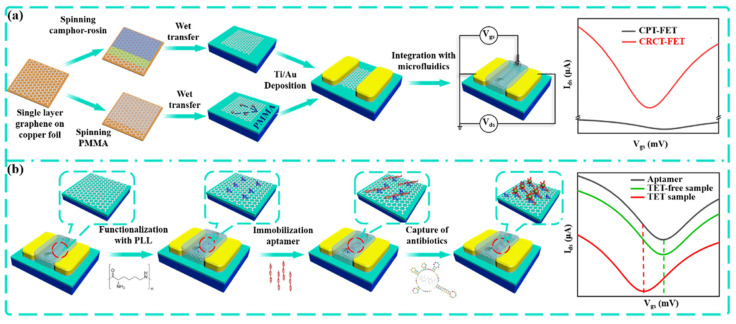
Schematic diagram of (**a**) graphene FET construction and (**b**) the fabrication process for the biosensor [53]. Reprinted with permission from [53]. Copyright 2022 American Chemical Society.

A biosensor based on an aptamer-functionalized field-effect transistor (FET) was developed by Wang et al. to detect tetracycline at ultra-low concentrations in seawater [53]. In order to improve the carrier mobility, the newly designed method for graphene FET preparation, so-called camphor−rosin clean transfer (CRCT), was used. Compared to the conventional transfer method, the CRCT-FET showed ten times higher carrier mobility, lower resistivity, and fewer defects. For the improvement of biosensor sensitivity, the inner surface of graphene FET (GFET) was functionalized with poly-L-lysine (PLL). Then, the aptamer (5′-CGT ACG GAA TTC GCT AGC CCC CCG GCA GGC CAC GGC TTG GGT TGG TCC CAC TGC GCG TGG ATC CGA GCT CCAC GTG-3′) was immobilized on PLL through electrostatic adsorption. Figure 10 shows the FET preparation process and the steps of its surface modification.

The operation of graphene FETs is based on changes of the Fermi level in the graphene channel (expressed by the Dirac voltage) via electrostatic gating triggered by specific adsorption of the analyte on its surface. The TET reaction with the aptamer affects the aptamer structure, which leads to a reduction in the hole density in the graphene channel and causes a change in the graphene doping level. This electrostatic gating effect leads to a shift in Dirac voltage, which is proportional to the concentration of TET. The developed aptasensor is characterized by exceptional sensitivity (with an LOD of 100 fM) and a wide linear range. The recovery rates obtained for spiked river and sea water ranged between 98% and 106%, which confirms the reliability of the CRCT-FET aptasensor in the analysis of environmental samples [53].

### 4.2. Soil

**Figure 11:**
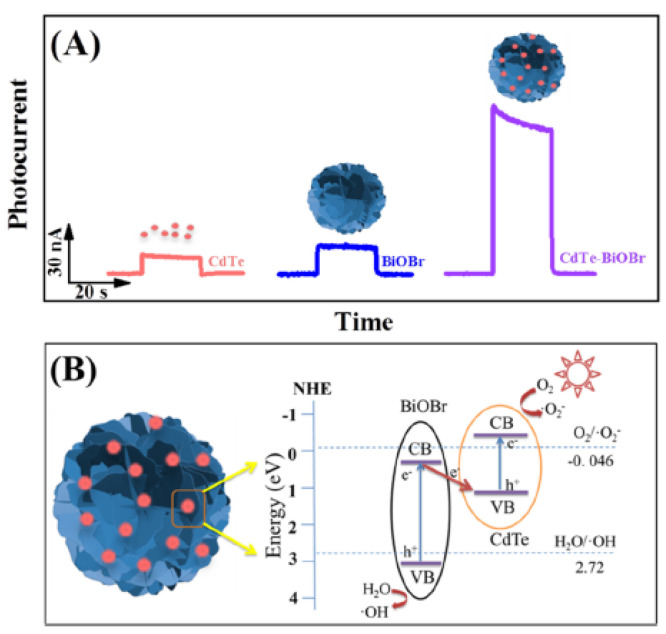
The mechanism of PEC of CdTe/ITO, BiOBr/ITO, and CdTe-BiOBr/ITO (**A**) and the Z-scheme-type electron transfer process of CdTe-BiOBr heterojunction (**B**) [54]. Reprinted from Ref. [54], copyright (2022), with permission from Elsevier.

For the determination of TET in soil, a photoelectrochemical aptasensor was developed [54]. As a photoactive material, bismuth oxobromide (BiOBr) in the form of nanoflowers was employed. It is characterized by a high efficiency of visible light utilization; however, its disadvantage is a fast recombination of photogenerated electron–hole pairs. To address this issue, the formation of a Z-scheme heterojunction by modification of BiOBr with CdTe quantum dots was proposed. The several-fold amplified intensity of the photocurrent of the CdTe-BiOBr heterojunction was the result of the low electron–hole combination efficiency, high visible light-utilization efficiency, and high carrier density of the heterojunction. The PEC performance of ITO electrodes modified with different materials and the Z-scheme-type electron transfer process of CdTe-BiOBr heterojunction are shown in Figure 11A,B, respectively.

To construct the aptasensor, the surface of the ITO electrode was modified by CdTe-BiOBr heterojunction synthesized by a hydrothermal method, and then the solution of the aptamer (5′-NH_2_-(CH_2_)_6_-CGT ACG GAA TTC GCT AGC CCC CCG GCA GGC CAC GGC TTG GGT TGG TCC CAC TGC GCG TGG ATC CGA GCT CCA CGT G-3′) was drop- casted. The aptamer/CdTe-BiOBr/ITO sensor exhibited a wide linear range (10–1500 pM) and a low detection limit of 9.25 pM. It showed good selectivity, examined during measurements in solutions containing, in addition to the analyte, other antibiotics: OTC, CTC, DOC, roxithromycin, ofloxacin, and sulfamethazine. To verify the analytical utility of the developed aptasensor, analyses of soil extracts were carried out. Soil samples were obtained from household farmland using manure fertilizer, and the extracts were obtained by shaking, sonicating, and centrifuging 1 g of soil treated with the buffer solution of pH 4.27 (selected by the authors on the basis of previous research). The recoveries of spiked soil extracts received in the range of 96–102% with an RSD of 1.16–7.10% indicated good accuracy and precision of the determinations. Analyses of the extracts obtained from the TET-enriched soil sample were also carried out. A recovery value of 73% was in good agreement with the result of the chromatographic analysis with a diode array detector (HPLC-DAD), indicating the low efficiency of the extraction procedure used.

In addition to the above-described examples, other (bio)sensors utilized for the determination of TCs in environmental samples are summarized in Table 2.

**Table 2:** Electrochemical (bio)sensors used for the detection of tetracyclines in environmental samples.

Sample	Type of Sensor	Analyte	Electrode Configuration	Electroanalytical Assay	Detection Method	LOD	Linear Range	Ref.
Tap water	Aptasensor	OXY DOX TET	dsDNA/CTAB-PNC/GCE	Decrease in ECL intensity	ECL	27 nM	0.1–10 µM	[49]
Water	Amperometric	TET	GCE/Zr-UiO-66/MWCNTs/AuNPs	Oxidation of TET	Ampero-metry	0.17 µM	0.5–225 µM	[50]
Sea water	Aptasensor	TET	Apt/PLL/GFET	Shift in Dirac voltage due to the TET-aptamer interaction	FET	100 fM	10 pM–100 nM	[53]
Soil	Aptasensor	TET	Apt/CdTe-BiOBr/ITO	Increase in photocurrent	PEC	9.25 ppm	10–1 500 ppm	[54]
Water	FET	TET	BP_Ag(+)_/MC/MIPs	Increase in relative resistance of the sensor	FET	7.94 nM	10 nM–1.0 µM	[55]
Lake water	Photoelectrochemical	TET	Ti_3_O/R-TiO_2_-700/Ti	Decrease in photocurrent	PEC	0.04 µM	3–300 µM	[56]
Waste water	Voltammetric	TET	Ag@Gum Arabic/C-SPE	Oxidation of TET	SWV	0.056 nM	0.1–1 250 nM	[57]
Tap, river water	Voltammetric	TET	N-CQD/GCE	Oxidation of TET	DPV	0.80 nM	5–30 nM	[58]
Tap, lake water	Amperometric	TET	ErGO/Cu-MOF/PtNPs/GCE	Oxidation of TET	Ampero-metry	0.03 µM	1.0–200 µM	[59]
Soil, lake water	Voltammetric	TET	DSMIP@Mn_3_O_4/_IL@rGO-MWCNT/GCE	Oxidation of TET	DPV	5 nM	0.01–20 µM	[60]

Detection method: ECL—electrochemiluminescence, FET—field-effect transistor detector, PEC—photoelectrochemical detection, SWV—square-wave voltammetry, DPV—differential pulse voltammetry. Electrode configuration: dsDNA—double-stranded DNA, CTAB—cetyltrimethylammonium bromide, PNC—perovskite nanocrystal, GCE—glassy carbon electrode, MWCNTs—multi-walled carbon nanotubes, AuNPs—gold nanoparticles, Apt—aptamer, PLL—poly-L-lysine, GFET—graphene field-effect transistor, ITO—indium tin oxide, BP—black phosphorus, MC—melamine cyanurate, /MIPs—molecularly imprinted polymers, C-SPE—carbon screen-printed electrode, N-CQD—nitrogen-doped carbon dots, ErGO—electrochemically reduced graphene oxide, Cu-MOF—copper metal–organic framework, PtNPS—platinum nanoparticles, IL—ionic liquid, rGO—reduced graphene oxide.

## 5. Recent Reports on Electrochemical (Bio)Sensors Designated for TCs Determination in Biological Samples

Benvidi et al. [61] presented an impedimetric aptasensor for TET detection in human serum samples. The GCE with deposited graphene oxide (GO) and chitosan (Chit) was utilized as a platform for immobilizing the aptamer (5′-NH2-CGT ACG GAA TTC GCT AGC CCC CCG GCA GGC CAC GGC TTG GGT TGG TCC CAC TGC GCG TGG ATC CGA GCT CCA CGT G-3′). To enhance the binding efficiency of the biorecognition element, a 1-pyrenebutyric acid-N-hydroxysuccinimide ester (Pyr) solution was applied on the electrode surface for 30 min before aptamer immobilization. Then, Pyr/Chit/GO/GCE was incubated with Apt solution for 6 h. The electrochemical impedance spectroscopy (EIS) was utilized to measure the changes in electron charge transfer resistance, which resulted from the binding of TET to the Apt/Pyr/Chit/GO/GCE surface. The measurements were performed in the presence of [Fe(CN)_6_]^4−/3−^ redox probe. The proposed biosensor demonstrated exceptional sensitivity, with an LOD equal to 0.32 fM, and good selectivity, even in the presence of other antibiotics from TCs, like OXY and DOX. The serum sample was diluted with PBS and then spiked with two concentrations of TET. The sensor’s effectiveness was confirmed by the determination of the antibiotic in human serum and pharmaceutical samples with good accuracy [61].

The utility of combining a molecularly imprinted polymer with multi-walled carbon nanotubes (MWCNTs) and L-histidine was presented in the work of Sulym et al. [62]. The sensor, based on a glassy carbon electrode, was suitably modified to determine TET in human serum, pharmaceuticals, and tap water samples. The surface modification of the sensor involved several synthesis steps. Physical adsorption was used to decorate PDMS (Polydimethylsiloxane) with MWCNTs. The mixture obtained in this way was activated in a solution of MES buffer with 1-ethyl-3-(3-dimethylaminopropyl)carbodiimide (EDC) and N-hydroxysuccinimide (NHS). In the next step, the activated mixture was combined with L-histidine and TET to create the molecular imprint. After applying the prepared modifying mixture on the GCE surface, the tetracycline molecules were removed using an acetic acid solution by incubation. Apart from the electrochemical characterization of the sensor by means of CV and EIS techniques, the measurements included the determination of analytical parameters, such as linearity, using DPV. For both standard solutions and serum samples with the addition of TET, separate curves of the dependence of ΔI on the concentration of tetracycline were determined. ΔI was calculated as the difference in currents measured after removal and after rebinding of the analyte. Therefore, the obtained DPV voltammograms show the decrease in the measured current with increasing TET concentrations. In optimized conditions, the linearity range was obtained within the limits of 10^−11^ M and 10^−10^ M, and LOD equaled to 2.642 × 10^−12^ M. The practical use of the sensor was checked for three completely different matrices: capsule dosage form, serum, and tap water. A carefully weighed amount of the pharmaceutical was crushed, dissolved in water, sonicated, and finally filtered. The previously frozen serum samples were thawed, tetracycline and acetonitrile were added, and then the mixture was vortexed. The sample was additionally centrifuged to settle any residual proteins. The tap water samples did not require any preparation. The standard addition method was used for each of the tested matrices to estimate recovery. For the pharmaceutical samples containing tetracycline, the accuracy error was less than 0.5%, with a repeatability error of below 2%. The recovery was 99.69, 98.92, and 100.60% for capsules, serum, and water, respectively. Both the accuracy and repeatability errors (RSD, *n* = 3) did not exceed 2%.

Some further examples of electrochemical (bio)sensors for TCs detection in biological and pharmaceutical samples, as well as sensors enabling the determination of this antibiotic in matrices of different origin, are described in Table 3.

## 6. Summary

In this review, the reports on electrochemical sensors and biosensors designed for the determination of antibiotics of the tetracycline group, published from 2022 to the present, were reviewed. The conducted literature survey indicated that the majority of reports pertain to analyses of food products of animal origin, mostly milk, but also honey and chicken meat. Far fewer reports dealt with analytical solutions dedicated to environmental analyses, and only two referred to biological samples.

The applicability of the proposed developments for analysis of a particular type of sample should be considered from two aspects: analytical characteristics of the sensor (for food analysis, especially, the limit of detection, which is important with respect to regulations relating to permitted antibiotic contents/concentrations) and sample preparation procedures.

Looking at the analytical parameters of the proposed methodologies, they should be considered at least satisfactory. All reported (bio)sensors allowed TCs to be determined at levels significantly below WHO and EU regulatory limits. For one of the elaborated aptasensors, BSA/KAP/MWCNTs-COOH/CNFs/GCE, the determined LOD value was extremely low, at 2.28 aM [24]. Developed (bio)sensors showed good selectivity, tested in particular towards antibiotics both from the tetracycline group and other classes of antibiotics. Extremely high selectivity, and even specificity, was characteristic of the aptasensors and those using MIPs. The accuracy of the proposed approaches was estimated based on the recovery values. Two approaches were used in this aspect: either the sample to be analyzed was spiked directly, which was then subjected to appropriate preparation, or the sample was prepared and then, at the final stage, enriched with the analyte. For obvious reasons, the first approach gives more reliable results. Regardless of the sample enrichment step with the analyte, the recoveries obtained were satisfactory and ranged, for the vast majority of biosensors, between 91% and 114%.

As for the preparation of samples for analysis, the easiest steps were required for samples of tap water, river water, and lake water. In most cases, they only required centrifugation and filtration, possibly preceded by sonication. The pretreatment of milk samples was more complex and involved precipitation of proteins and fat, for example, with ethanol [24,32], trichloroacetic acid [26], or perchloric acid [28], then filtration and/or centrifugation. Honey samples were either only diluted [34] or subjected to a similar procedure as described above for milk [35]. Chicken meat samples were minced, then protein and fat were precipitated and separated, analogously to milk samples [36,37]. Other sample pretreatment was proposed for the use of the aptasensor designated for the analysis of river water and milk [49]; both types of samples were subjected to the same preparation procedure, including filtration, addition of ethyl acetate and sodium chloride, vortexing, centrifugation, drying the supernatant under nitrogen, and finally, dissolving the solid residue in ultrapure water. To remove proteins from human serum, frozen serum samples were thawed, treated with acetonitrile, and then centrifuged [60]. It is worth noting that only a few approaches have been proposed in which sample preparation is limited to simple and quick steps, such as diluting with a buffer in the case of milk [31], honey [34], and human serum [59], filtering and centrifuging of milk [27], and grinding of chicken ham in a commercial blender [38]. In several cases, the authors did not describe an important step in the analytical procedure, which is sample pretreatment.

**Table 3:** Electrochemical (bio)sensors used for the detection of tetracyclines in matrices of different origin.

Sample	Type of Sensor	Analyte	Electrode Configuration	Electroanalytical Assay	Detection Method	LOD	Linear Range	Ref.
Tablets, serum	Aptasensor	TET	Apt/Pyr/Chit/GO/GCE	The change in electron charge transfer resistance	EIS	0.32 fM	1.0 fM–316 nM	[61]
Tablets, serum, tap water	Voltammetric	TET	L-His-MWCNTs@PDMS-5/MIP	Difference in current after removal and rebinding of TET	DPV	2.64 pM	0.01–0.1 nM	[62]
Tablets, milk	Voltammetric	TET	Au@MoS_2_/Ch/GCE	Oxidation of TET	DPV	0.41 µM	1–1000 µM	[63]
Human serum, water	Voltammetric	TET	RGO-ZnO/GCE	Oxidation of TET	SWV	0.38 µM	4–400 µM	[64]
Tablets, milk, honey, lake water	ECL sensor	TET	MIP/NUZ@Ru/GMI/GCE	Decrease in ECL intensity	ECL	20 nM	0.05–1000 µM	[65]
Milk, cosmetic lotion	Enzymatic biosensor	TET	SPE/Chit/MWCNTs/AuNPs/TYR SPE/Chit/RGO/AuNPs/TYR	Decrease in enzymatic phenol oxidation peak current due to inhibition by TET	Amperometry	15 pM 0.2 nM	1 nM–10 µM 0.1 nM–10 µM	[33]
Milk, river water	Photoelectrochemical	TET	ZIF-8@ZIS/FTO	Decrease in fluorescence due to TET-ZIF-8 interaction	PEC	0.1 pM	1.0 pM–100 nM	[35]
Honey, water	Single-chamber microbial fuel cells	TET	A(-): CP/PEO-NFs K(+): PTFE/CP/Pt-C	Decrease in current density	Current dependent on the metabolic activity of microorganism	-	-	[36]
Milk, chicken, water	Voltammetric	TET	SPE/Fe_3_O_4_@COFs@MIPs	Oxidation of TET	DPV	-	0.23–225 µM	[37]
Milk, chicken, water	Aptasensor	TET	Fe_3_O_4_-Apt/cDNA-MOF-MB	Fluorescence of MOF and oxidation of methylene blue	Dual mode: fluorescence and DPV	0.38 µM 0.26 µM	2.25 µM–225 mM	[41]
Milk, honey, black tea, tap, pond, river water	Aptasensor	TET	Apt/WO_3_@CNTs@AuNPs/GCE	Oxidation of [Fe(CN)_6_]^4−/3−^	DPV	0.048 nM	0.1–100 nM	[48]
Milk, river water	cDNA biosensor	TET	MCH/cDNA/Ag@AgPOM/GCE	Decrease in ECL intensity	ECL	0.14 fM	0.2 fM–10 mM	[51]
Milk, river samples	Aptasensor	TET	BSA-Apt-AuNPs@MoS_2_NS/GCE	Oxidation of MB and fluorescence of ssDNA-MB@AuNPs@COF	Dual mode: DPV and fluorescence	0.03 nM 0.05 nM	10 nM–10 mM	[52]
Milk, river water	Photoelectrochemical	TET	MIP/Au-ZIS/ITO	Dual signal: anode and cathode photocurrent	Dual PEC	0.82 nM 0.67 nM	1 nM–0.5 mM	[66]
Milk, lake water	Photoelectrochemical	TET	CB-Co{P_4_Mo_6_}_2_-Nafion/GCE	Increase in oxidation peak current with TET concentration under exposure to light	DPV	5.2 nM	0.1–1.0 µM	[67]
Honey, river water	Aptasensor	TET	Apt/TiO_2_-Ag/NDG/ITO	Decrease in cathodic photocurrent	PEC	30 pM	100 pM–100 nM	[68]
Milk, lake water	Photoelectrochemical	TET	CB-{Co_16_Mo_16_P_24_}-Nafion/GCE	Increase in oxidation peak current with TET concentration under exposure to light	DPV	33.45 nM	0.1–1.0 µM	[69]
Honey, tap, mineral water	Voltammetric	TET	MnO_2_/GCE	Oxidation of TET	LSV	0.51 µM	1.0–4.0 µM	[70]
**Electrochemical (bio)sensors not used for the analysis of real samples**								
-	Aptasensor	TET	MB-Apt/AuE	Oxidation of methylene blue	DPV	1.2 nM	1 nM–1 µM	[31]
-	Voltammetric	TET	Fe-Mt-150/GCE	Oxidation of TET	CV	-	-	[71]
-	Voltammetric	TET	MnWO_4_/f-CMF/GCE	Oxidation of TET	DPV	0.24 µM	1.75–409.25 µM	[72]
-	Photoelectrochemical	TET	g-C_3_N_4_/TiO_2_@CC	Decrease in photocurrent	PEC	0.42 pM	1–100 µM	[73]
-	Voltammetric	TET	MIP-GH-NSCMS/GCE	Difference in current after removal and rebinding of TET	DPV	50 nM	0.1–50 µM	[74]

Detection method: EIS—electrochemical impedance spectroscopy, DPV—differential pulse voltammetry, SWV—square-wave voltammetry, ECL—electrochemiluminescence, PEC—photoelectrochemical detection, LSV—linear sweep voltammetry, CV—cyclic voltammetry. Electrode configuration: Apt—aptamer, Pyr—1-pyrenebutyric acid-N-hydroxysuccinimide ester, Chit or Ch—chitosan, GO—graphene oxide, GCE—glassy carbon electrode, MWCNTs—multi-walled carbon nanotubes, PDMS-5—polydimethylsiloxane, MIP—molecularly imprinted polymer, RGO—reduced graphene oxide, GMI—graphene–carbon nanotube composite, SPE—screen-printed electrode, AuNPs—gold nanoparticles, TYR—tyrosinase, FTO—fluorine-doped tin oxide, CP—carbon paper, PEO—polyethylene oxide, NFs—nanofibers, PTFE—polytetrafluoroethylene, COFs—covalent organic frameworks, cDNA—capture DNA, MOF—metal–organic framework, MB—methylene blue, CNTs—carbon nanotubes, MCH—6-mercaptohexan-1-ol, POM—porphyrin metal−organic matrix, BSA—bovine serum albumin, ITO—indium tin oxide, CB—carbon black; NDG—nitrogen-doped graphene, AuE—gold electrode, f-CNF—functionalized carbon nanofibers, CC—carbon cloth, GH-NSCMS—N, S co-doped carbon microsphere/graphene composite hydrogel.

Among considered analytes, the vast majority of published reports focused on tetracycline determination. Several proposed developments allow for the determination of the sum content of antibiotics of the tetracycline group, such as TET, DOX, OXY, and AUR.

However, the use of a chemometric approach, in particular the principal component analysis (PCA) method, to analyze the measurement data recorded with the use of a chemiluminescent aptasensor in a solution containing TET, OXY, and DOX, allowed the differentiation of tetracyclines [47].

The most frequently reported mechanism of bioelectrochemical detection of TCs was sensing using aptamers. Surprisingly, only one developed aptasensor was tested for regeneration and reusability [28]. Out of all sensors, those employing MIPs to increase selectivity were most often reported. In order to improve the analytical characteristics of the elaborated (bio)sensors, the electrode composites were modified with metal–organic frameworks (MOFs) in addition to conductivity-enhancing components such as carbon nanotubes (CNTs) and gold nanoparticles (AuNPs). Among the detection techniques employed, amperometry dominates; nevertheless, photoelectrochemical, electrochemiluminescence, and electrochemical/fluorescence dual-mode detections have also been applied. A few potentiometric sensors have also been developed for the determination of TCs.

## 7. Future Perspective

Sensors and biosensors are analytical tools that have been under intensive development for several years. The reason for that is the number of advantages they have. This includes the possibility of analyzing samples without the time-consuming step of sample pretreatment, miniaturization, and automatization of the analysis process, promoting lower consumption of reagents and energy required for analysis, and therefore they are in line with the trends of not only green analytical chemistry but also, in a broader context, sustainable development. Among the group of (bio)sensors that are of great interest to scientists and companies implementing new technologies are those that use electrochemical transducers. This is due to the easy miniaturization of detection devices, their low price, and the possibility of constructing efficient analytical tools for on-site analysis.

Further development in bioelectrochemical-sensing analytical methodologies towards antibiotics detection will most likely concern environmental and food analysis.

Due to the relatively simple pretreatment of environmental samples, the on-site verification of the state of the environment by means of sensors appears to be achievable, although solutions for portable devices operating in autonomous mode should be proposed. In this regard, research is needed into sensors whose receptors can be regenerated and reused for analysis. Moreover, it would be beneficial to develop sensing platforms for multi-component analyses, for example, for the determination of not only tetracyclines but also other contaminating drugs.

The analysis of food products for pharmaceuticals appears to be another wide area for the use of biosensors in the future. An intensive development of electrochemical approaches dedicated to food analysis may be related to the growing public awareness of the widespread use of antibiotics in animal husbandry and thus the need for quick and simple analytical procedures to verify the quality of food products. However, complex matrices of food products containing, e.g., proteins and fat, require pretreatment of samples, thus, on-site analysis appears to be limited. Research should therefore be undertaken into sensors whose use would require simple sample preparation. This also involves the development of receptor layers that can be regenerated, as fats and proteins, even in small amounts, can cause electrode fouling and block the receptor surface. Aptasensors appear to be preferable for this purpose because of their ability to regenerate. A big advantage of aptamers is also their exceptional selectivity. It seems that future trends in this field will focus on the development of new aptasensors designed for a variety of analytes, not only antibiotics from the tetracycline group.

Regardless of the type of sample analyzed, key features enabling (bio)sensors to become an efficient tool for analysis in the future are analytical effectiveness, easy sample pretreatment, stability over long-term usage, user-friendliness, and portability. Therefore, further research should be carried out taking these guidelines into account.

## Data Availability

Not applicable.
